# Supplementary material for: N-hydroxypipecolic acid triggers systemic acquired resistance through extracellular NAD(P)
Source: Nat Commun. 2023 Oct 27;14:6848. doi: 10.1038/s41467-023-42629-0 (PMC10611778; doi:10.1038/s41467-023-42629-0)
Supplement: Supplementary file 1 — Supplementary Information [file 41467_2023_42629_MOESM1_ESM.pdf]

Supplementary Information

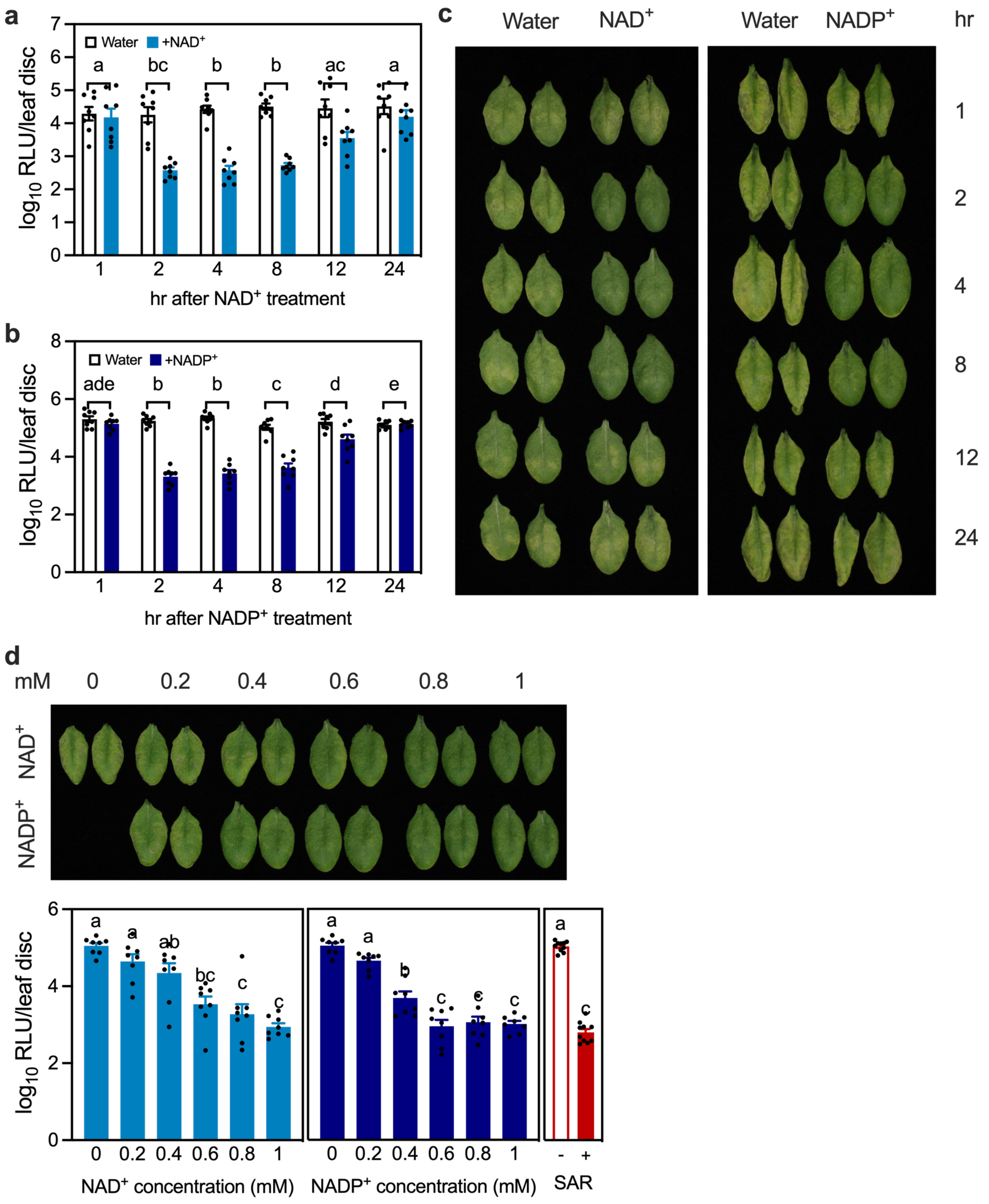

Supplementary Figure 1 Exogenous NAD(P)$^+$ rapidly induces robust short-duration systemic immunity

a, **b**, The dynamics of $NAD(P)^+$-induced systemic immunity in wild-type plants. Three lower leaves on each four-week-old plant were infiltrated with 1 mM $NAD(P)^+$ or water. At the indicated times, one upper systemic leaf on the plant was inoculated with *Psm*. Samples were collected 72 hr post inoculation (hpi). RLU: relative light unit. Bars represent means ± SE (n = 8 independent leaf disks). Different letters denote significant differences (two-way ANOVA with Tukey's test; p values are shown in the Source Data file).

c, Disease symptoms on the inoculated systemic leaves at 72 hpi in (**a** and **b**).

d, Systemic immunity induced by different concentrations of $NAD(P)^+$ in wild-type plants with the systemic leaves being inoculated at 4 hr after the $NAD(P)^+$ treatment. For biological induction of SAR, three lower leaves on each plant were infiltrated with 1 mM $MgCl_2$ (-SAR) or *Psm* (+SAR). Two days later, one upper systemic leaf on the plant was inoculated with *Psm*. Samples were collected 72 hpi. Bars represent means ± SE (n = 8 independent leaf disks for the $NAD(P)^+$ treatments and n = 10 independent leaf disks for the SAR assay). Different letters denote significant differences (one-way ANOVA with Tukey's test; p values are shown in the Source Data file).

Experiments in (**a**), (**b**), and (**d**) were conducted three times with similar results.

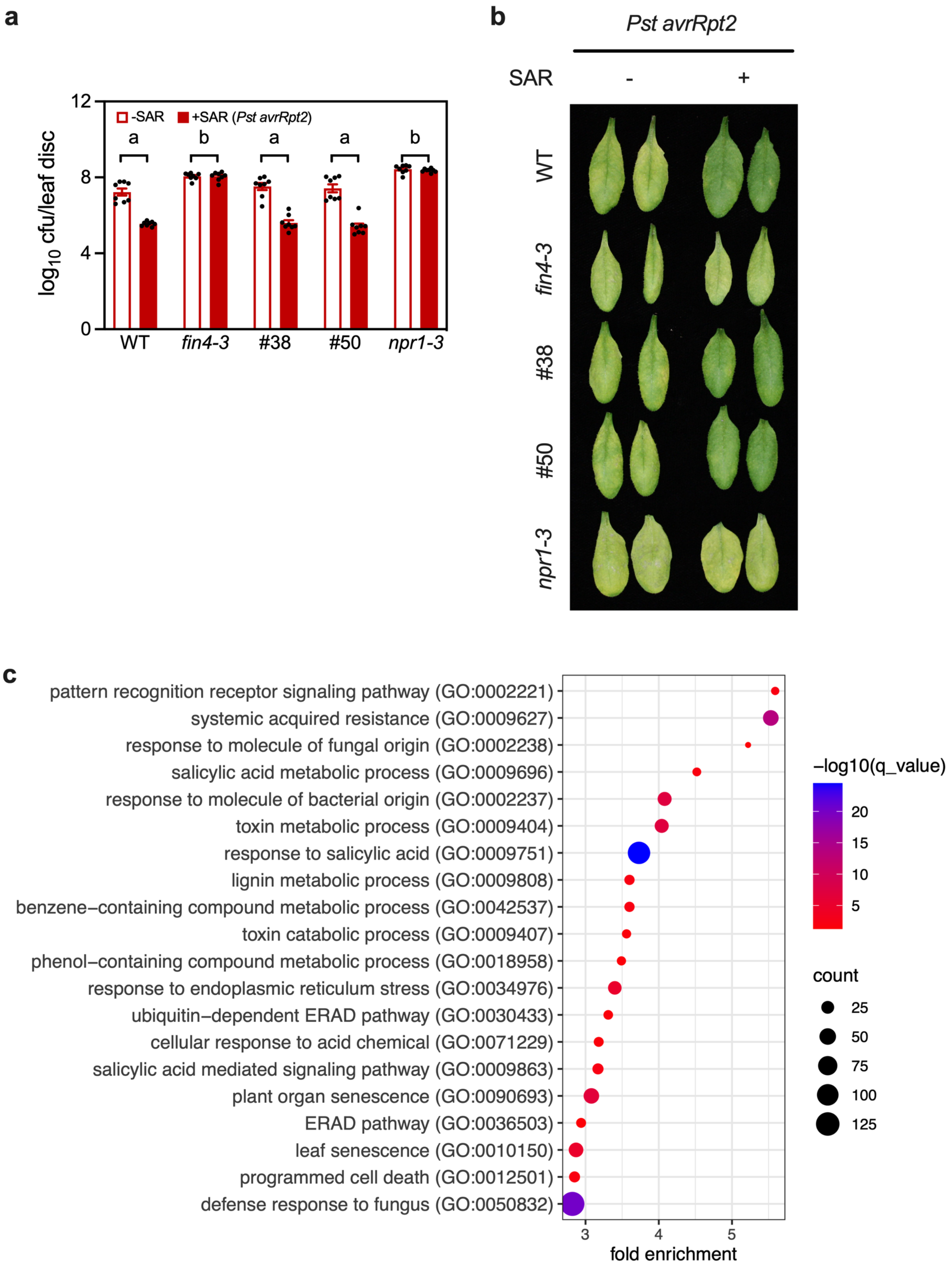

Supplementary Figure 2 Induction of SAR in *fin4-3* by *Pst avrRpt2* and pathways downregulated in the systemic leaves of *fin4-3*

a, Biological induction of SAR with the avirulent bacterial pathogen *Pst avrRpt2* in wild type (WT), *fin4-3*, two *35S:FIN4/fin4-3* complementation lines, and *npr1-3*. Three lower leaves on each four-week-old plant were infiltrated with *Pst avrRpt2* (OD_{600} = 0.002) (+SAR) or 1 mM $MgCl_2$ (-SAR). After 48 hr, one systemic leaf on each plant was inoculated with *Psm* (OD_{600} = 0.001). Samples from the inoculated systemic leaves were collected 72 hr post inoculation (hpi). Bars represent means ± SE (n = 8 independent leaf disks). Different letters denote significant differences (two-way ANOVA with Sidak's test; p values are shown in the Source Data file). The experiment was conducted three times with similar results.

b, Disease symptoms on the inoculated systemic leaves in (**a**). The photo was taken 72 hpi. SAR was induced with the avirulent pathogen *Pst avrRpt2*.

c, Gene ontology (GO) enrichment analysis of the 1,924 DEGs (Supplementary Data 1) identified in the systemic leaves of wild-type and *fin4-3* plants at 48 hr after SAR induction. The top 20 significantly enriched GO terms are listed.

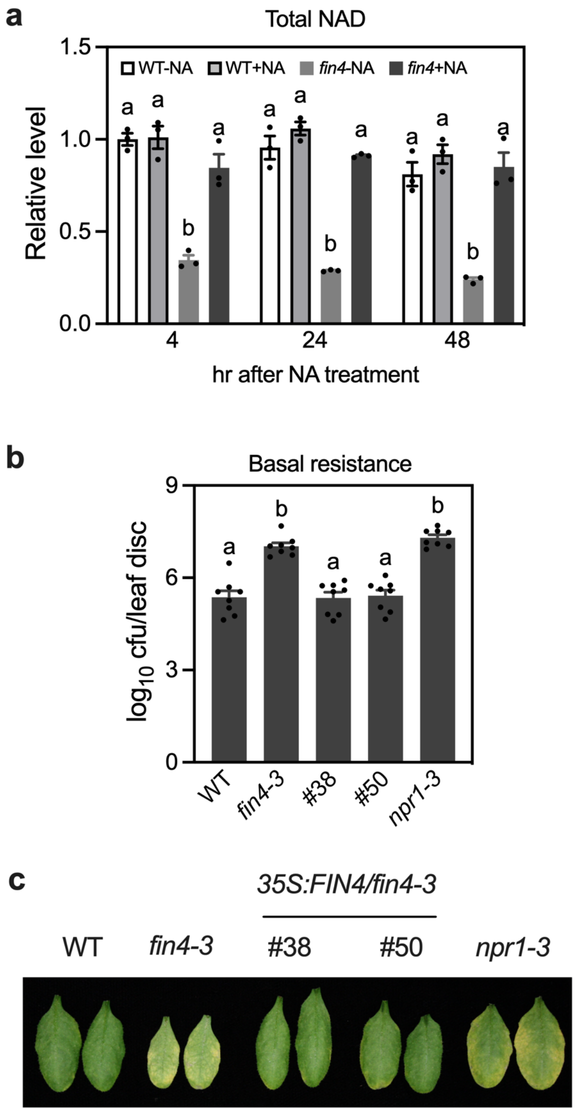

Supplementary Figure 3 Restoration of intracellular NAD in *fin4-3* by NA and compromised basal resistance in *fin4-3*

a, NA quickly restores intracellular NAD levels in *fin4-3*. Three leaves on each wild-type (WT) or *fin4-3* (*fin4*) plant were infiltrated with 4 mM NA. The infiltrated leaves were collected at the indicated times for total NAD measurement. Values are expressed relative to the NAD level in WT at 4 hr, which was arbitrarily set to 1. Bars represent means ± SE (n = 3 independent leaf samples). Different letters denote significant differences (one-way ANOVA with Tukey's test; p

values are shown in the Source Data file). NA did not change NAD levels in WT but restored NAD in *fin4-3* to wild-type levels from 4 to 48 hr after the treatment.

b, Basal resistance in WT, *fin4-3*, two *35S:FIN4/fin4-3* complementation lines, and *npr1-3*. Three leaves on each four-week-old plant were inoculated with *Psm* (OD_{600} = 0.0001). Samples were collected 72 hpi. Bars represent means ± SE (n = 8 independent leaf disks). Different letters denote significant differences (one-way ANOVA with Tukey’s test; p values are shown in the Source Data file). The *fin4-3* mutant exhibited enhanced susceptibility to *Psm*. The experiment was conducted three times with similar results.

c, Disease symptoms on the inoculated leaves in (**b**). The photo was taken 72 hpi.

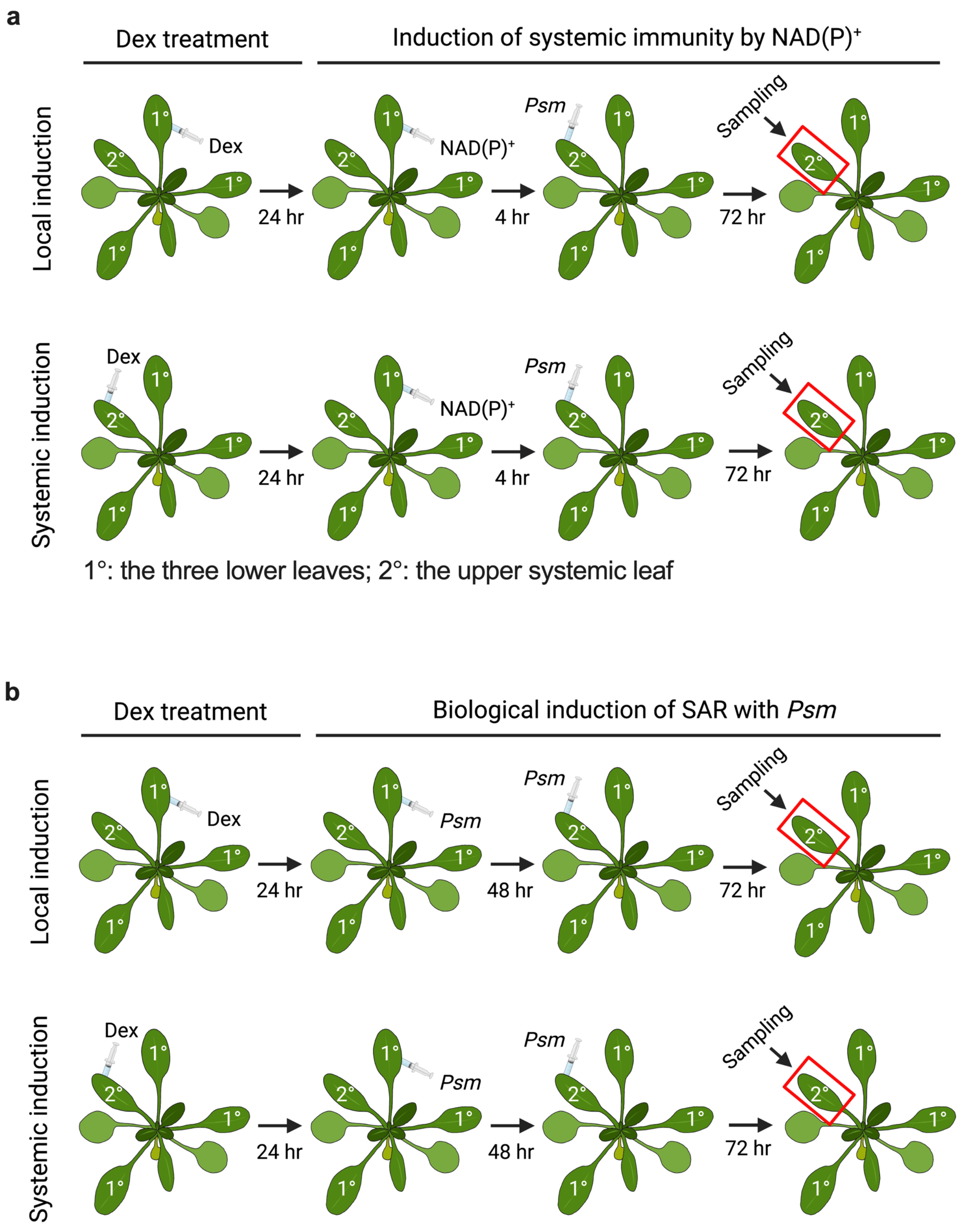

Supplementary Figure 4 Schematic diagrams of Dex application

a, **b**, Schematic diagrams of testing the effects of local and systemic induction of *LecRK-VI.2* on NAD(P)$^+$-induced systemic immunity (**a**) and biological induction of SAR (**b**) in *Dex:LecRK-VI.2/lecrk-VI.2* plants.

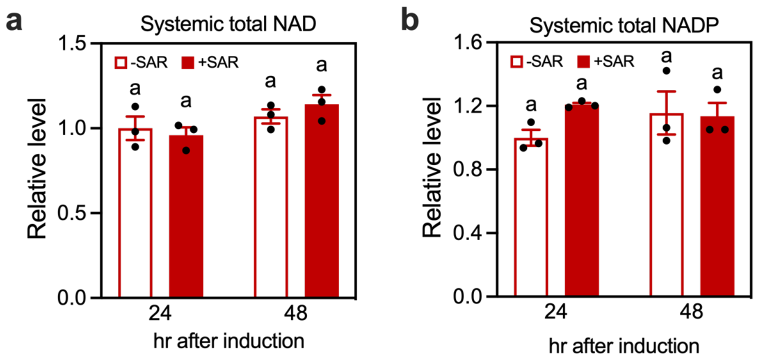

Supplementary Figure 5 Total NAD(P) levels in the systemic leaves

a, **b**, SAR induction does not significantly change total NAD(P) levels in systemic leaves. Three lower leaves on each wild-type plant were infiltrated with *Psm* (OD_{600} = 0.002) (+SAR) or 1 mM $MgCl_2$ (-SAR). One systemic leaf on each plant was collected at the indicated times for total NAD(P) measurement. Values are expressed relative to the NAD(P) levels in -SAR samples at 24 hr, which were arbitrarily set to 1. Bars represent means ± SE (n = 3 independent leaf samples). Same letters denote no significant differences (one-way ANOVA with Tukey's test; p values are shown in the Source Data file). The experiment was repeated with similar results.

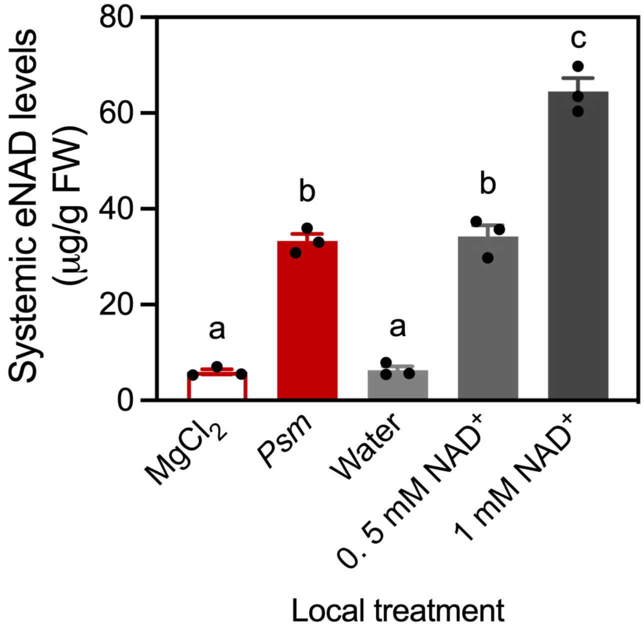

Supplementary Figure 6 Comparison of systemic eNAD levels upon SAR induction and NAD^+ treatment

Three lower leaves on each wild-type plant were infiltrated with 1 mM $MgCl_2$, *Psm* (OD_{600} = 0.002), water, 0.5 mM NAD^+, or 1 mM NAD^+. One systemic leaf on each plant was collected at 4 hr after the water and NAD^+ treatments and 48 hr after the $MgCl_2$ and *Psm* treatments for AWF extraction and eNAD measurement. The eNAD levels were calculated to μg/g fresh weight (FW) based on a standard curve. Bars represent means ± SE (n = 3 independent AWF samples). Different letters denote significant differences (one-way ANOVA with Tukey's test; p values are shown in the Source Data file). The experiment was repeated with similar result.

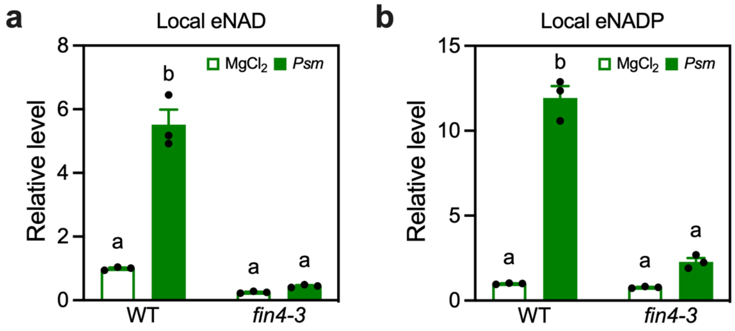

Supplementary Figure 7 Pathogen-induced local accumulation of eNAD(P) in *fin4-3*

a, b, Three leaves on each plant were infiltrated with 1 mM $MgCl_2$ or *Psm* (OD_{600} = 0.002). The infiltrated leaves were collected 24 hr later for AWF extraction and eNAD(P) measurement. Values are expressed relative to the eNAD(P) levels in the wild type (WT) treated with $MgCl_2$, which were arbitrarily set to 1. Bars represent means ± SE (n = 3 independent AWF samples). Different letters denote significant differences (one-way ANOVA with Tukey's test; p values are shown in the Source Data file). The experiment was repeated with similar results.

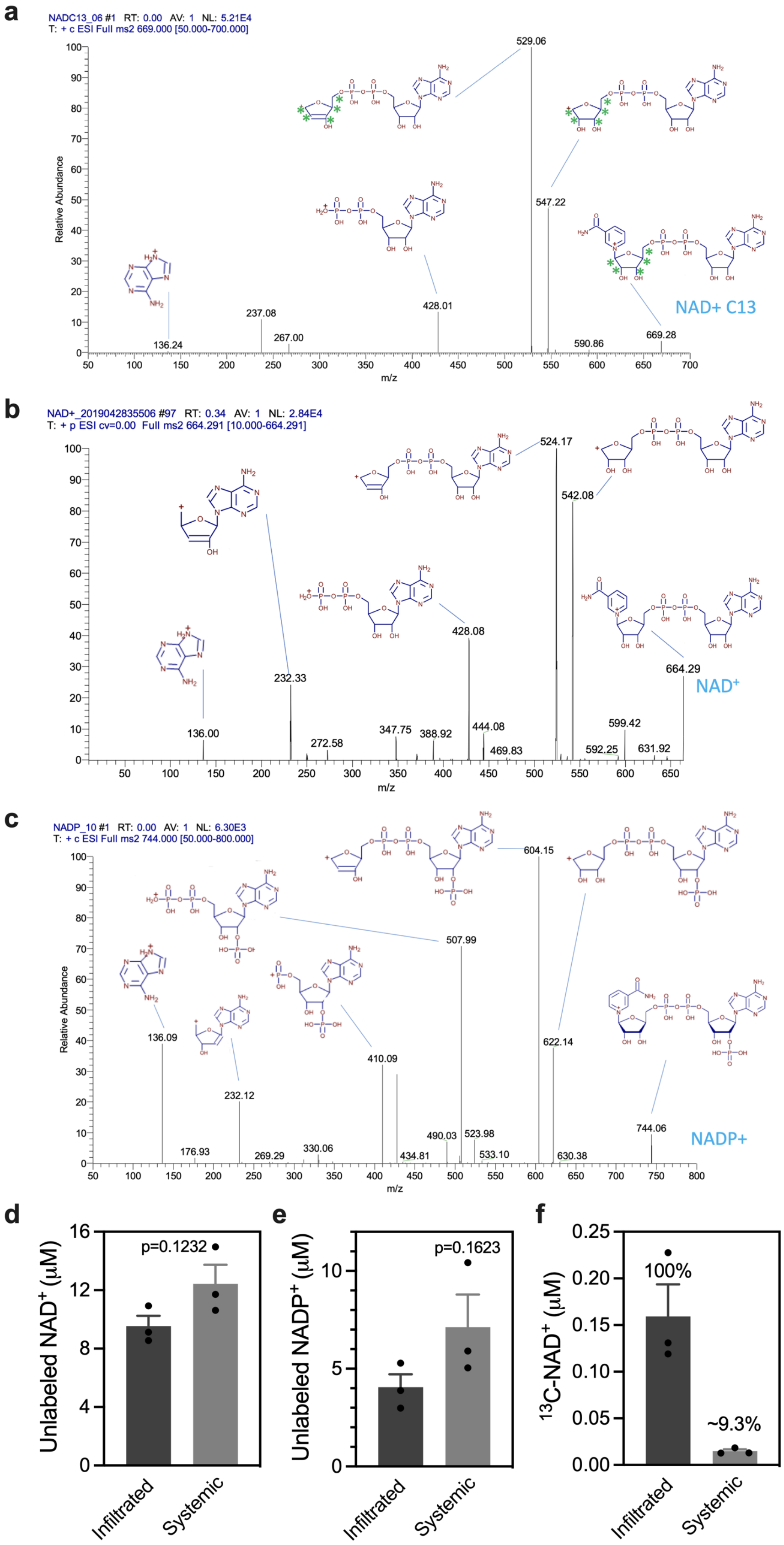

a
NADC13_06 #1 RT: 0.00 AV: 1 NL: 5.21E4
T: + c ESI Full ms2 669.000 [50.000-700.000]
Relative Abundance
529.06
547.22
428.01
237.08
267.00
136.24
590.86
669.28
NAD+ C13
m/z
b
NAD+_2019042835506 #97 RT: 0.34 AV: 1 NL: 2.84E4
T: + p ESI cv=0.00 Full ms2 664.291 [10.000-664.291]
524.17
542.08
428.08
232.33
136.00
664.29
NAD+
347.75
388.92
444.08
469.83
272.58
592.25
599.42
631.92
m/z
c
NADP_10 #1 RT: 0.00 AV: 1 NL: 6.30E3
T: + c ESI Full ms2 744.000 [50.000-800.000]
Relative Abundance
604.15
507.99
622.14
410.09
136.09
232.12
176.93
269.29
330.06
434.81
490.03
523.98
533.10
630.38
744.06
NADP+
m/z
d
Unlabeled NAD+ (μM)
p=0.1232
Infiltrated
Systemic
e
Unlabeled NADP+ (μM)
p=0.1623
Infiltrated
Systemic
f
13C-NAD+ (μM)
100%
~9.3%
Infiltrated
Systemic

Supplementary Figure 8 Exogenously added NAD^+ moves systemically in its intact form

a, MS/MS spectrum of ^{13}C-NAD^+.

b, MS/MS spectrum of NAD^+.

c, MS/MS spectrum of $NADP^+$.

d, Unlabeled NAD^+ levels in the infiltrated and upper noninfiltrated systemic leaf extracts are not significantly different (two-tailed t test). Bars represent means ± SE (n = 3 independent leaf extract samples).

e, Unlabeled $NADP^+$ levels in the infiltrated and upper noninfiltrated systemic leaf extracts are not significantly different (two-tailed t test). Bars represent means ± SE (n = 3 independent leaf extract samples).

f, ^{13}C-NAD^+ levels in the infiltrated and upper noninfiltrated systemic leaf extracts.

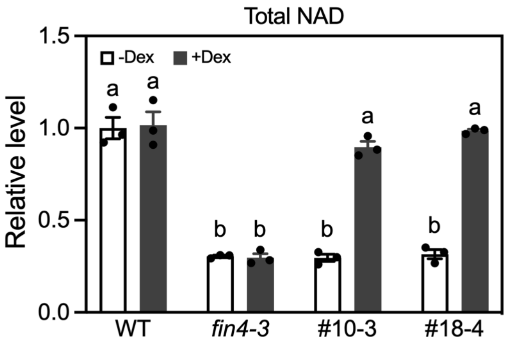

Supplementary Figure 9 Restoration of NAD levels in the *Dex:FIN4/fin4-3* plants by Dex application

Dex application restores NAD levels in the *Dex:FIN4/fin4-3* plants. Two leaves on each four-week-old wild-type (WT), *fin4-3*, and *Dex:FIN4/fin4-3* plants were infiltrated with 50 µM Dex (+Dex) or 0.1% methanol (-Dex). Twenty-four hr later, the infiltrated leaves were collected for total NAD measurement. Values are expressed relative to the NAD level in WT/-Dex samples,

which was arbitrarily set to 1. Bars represent means ± SE (n = 3 independent leaf samples). Different letters denote significant differences (one-way ANOVA with Tukey's test; p values are shown in the Source Data file). The experiment was repeated with similar results.

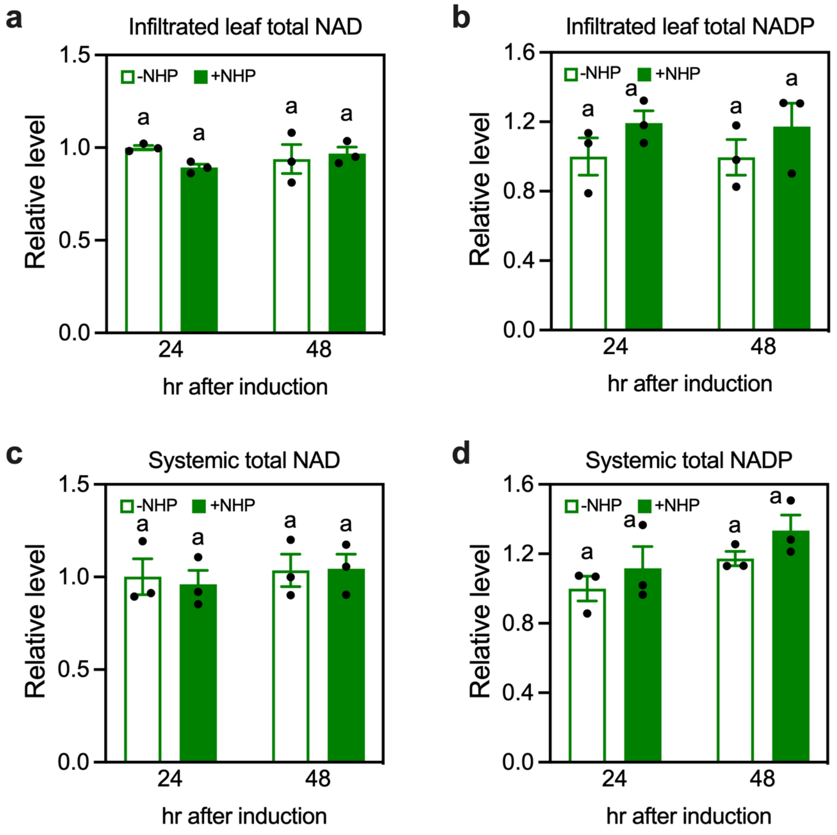

Supplementary Figure 10 Total NAD(P) levels in NHP-treated leaves and upper systemic leaves

a, **b**, Total NAD(P) levels in NHP-infiltrated leaves. Two leaves on each wild-type plant were infiltrated with 0.5 mM NHP or water (-NHP). The infiltrated leaves were collected at the indicated times for total NAD(P) measurement. Values are expressed relative to the NAD(P) levels in -NHP samples at 24 hr, which were arbitrarily set to 1. Bars represent means ± SE (n =

3 independent leaf samples). Same letters denote no significant differences (one-way ANOVA with Tukey's test; p values are shown in the Source Data file).

c, **d**, Total NAD(P) levels in upper systemic leaves on plants with lower leaves treated with NHP. Three lower leaves on each wild-type plant were infiltrated with 0.5 mM NHP or water. One systemic leaf on each plant was collected at the indicated times for total NAD(P) measurement. Values are expressed relative to the NAD(P) levels in -NHP samples at 24 hr, which were arbitrarily set to 1. Bars represent means ± SE (n = 3 independent leaf samples). Same letters denote no significant differences (one-way ANOVA with Tukey's test; p values are shown in the Source Data file).

The experiments in (**a**), (**b**), (**c**), and (**d**) were repeated with similar results.

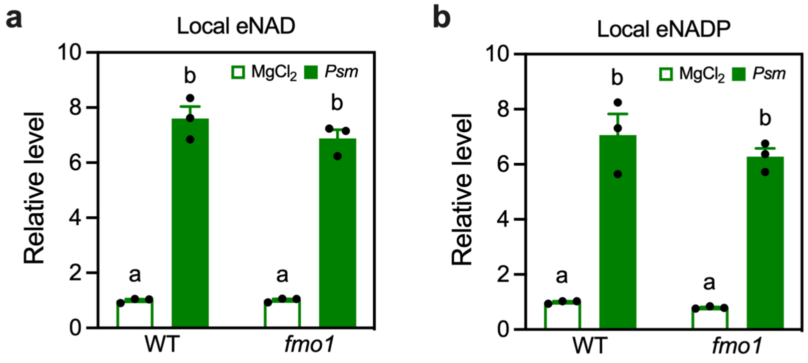

Supplementary Figure 11 Pathogen-induced local accumulation of eNAD(P) in *fmo1*

a, b, Three leaves on each plant were infiltrated with 1 mM $MgCl_2$ or *Psm* (OD_{600} = 0.002). The infiltrated leaves were collected 24 hr later for AWF extraction and eNAD(P) measurement. Values are expressed relative to the eNAD(P) levels in the wild type (WT) treated with $MgCl_2$, which were arbitrarily set to 1. Bars represent means ± SE (n = 3 independent AWF samples).

Different letters denote significant differences (one-way ANOVA with Tukey's test; p values are shown in the Source Data file). The experiment was repeated with similar results.

Supplementary Table 1 Oligos used for subcloning in this study

Oligo name	Sequence (5' to 3')
KpnI-FIN4F	GGGGTACCATGGCGGCTCATGTTTCTAC
XbaI-FIN4R	GCTCTAGATTAGCAATCAATAAGTGAGCTG
attB1LecRK-VI.2F	AAAAAGCAGGCTCACCCATGGGCACACAAAGATCCATG
attB2LecRK-VI.2R	AGAAAGCTGGGTCTACTGACTGATACGAGAAGTC
attB1FIN4F	AAAAAGCAGGCTCGGAAATGGCGGCTCATGTTTCTACTGGAAAC
attB2FIN4R	AGAAAGCTGGGTTTAGCAATCAATAAGTGAGCTGCTAC
