# Supplementary material for: N-hydroxypipecolic acid triggers systemic acquired resistance through extracellular NAD(P)
Source: Nat Commun. 2023 Oct 27;14:6848. doi: 10.1038/s41467-023-42629-0 (PMC10611778; doi:10.1038/s41467-023-42629-0)
Supplement: Supplementary file 3 — Reporting Summary [file 41467_2023_42629_MOESM3_ESM.pdf]

nature portfolio

Corresponding author(s): Zhonglin Mou

Last updated by author(s): 2023-10-6

Reporting Summary

Nature Portfolio wishes to improve the reproducibility of the work that we publish. This form provides structure for consistency and transparency in reporting. For further information on Nature Portfolio policies, see our Editorial Policies and the Editorial Policy Checklist.

Statistics

For all statistical analyses, confirm that the following items are present in the figure legend, table legend, main text, or Methods section.

n/a	Confirmed	
☐	☒	The exact sample size (*n*) for each experimental group/condition, given as a discrete number and unit of measurement
☐	☒	A statement on whether measurements were taken from distinct samples or whether the same sample was measured repeatedly
☐	☒	The statistical test(s) used AND whether they are one- or two-sided<br>*Only common tests should be described solely by name; describe more complex techniques in the Methods section.*
☒	☐	A description of all covariates tested
☒	☐	A description of any assumptions or corrections, such as tests of normality and adjustment for multiple comparisons
☒	☐	A full description of the statistical parameters including central tendency (e.g. means) or other basic estimates (e.g. regression coefficient) AND variation (e.g. standard deviation) or associated estimates of uncertainty (e.g. confidence intervals)
☒	☐	For null hypothesis testing, the test statistic (e.g. *F*, *t*, *r*) with confidence intervals, effect sizes, degrees of freedom and *P* value noted<br>*Give P values as exact values whenever suitable.*
☒	☐	For Bayesian analysis, information on the choice of priors and Markov chain Monte Carlo settings
☒	☐	For hierarchical and complex designs, identification of the appropriate level for tests and full reporting of outcomes
☐	☒	Estimates of effect sizes (e.g. Cohen's *d*, Pearson's *r*), indicating how they were calculated

Our web collection on statistics for biologists contains articles on many of the points above.

Software and code

Policy information about availability of computer code

Data collection	The Cutadapt package (v3.4) was used to clean reads acquired from Illumina NovaSeq 6000 platform for trimming adaptors and low-quality bases with a quality phred-like score <20. The STAR package (Spliced Transcripts Alignment to a Reference, v2.7.9a) was used to map the cleaned reads to the reference genome. The HTSeq (High-Throughput Sequence Analysis in Python, v0.11.2)62, samtools, and scripts developed in house at UF ICBR to process the mapping results to remove potential PCR duplicates and count uniquely mapped reads for gene expression analysis. Xcalibur 3.1 and Freestyle (Thermo Fisher Scientific) were used for quantification of the mass spectrometry peaks.
Data analysis	The R-package (v4.1.3) was used to perform PCA analysis and the DESeq2-based R pipeline was used to analyze the gene expression levels. Heatmap was made with TBtools. PANTHER version 14 was used to conduct GO analysis and R-package ggplot2 was used to create graphics. Microsoft Excel of Microsoft Office 2023 for Macintosh was used for Student's t test, and Prism 10 was used for one-way and two-way ANOVA analyses.

For manuscripts utilizing custom algorithms or software that are central to the research but not yet described in published literature, software must be made available to editors and reviewers. We strongly encourage code deposition in a community repository (e.g. GitHub). See the Nature Portfolio guidelines for submitting code & software for further information.

Data

Policy information about availability of data

All manuscripts must include a data availability statement. This statement should provide the following information, where applicable:

- Accession codes, unique identifiers, or web links for publicly available datasets
- A description of any restrictions on data availability
- For clinical datasets or third party data, please ensure that the statement adheres to our policy

The RNA-seq data that support the findings of this study have been deposited in the Gene Expression Omnibus database with the accession number GSE225107.

Research involving human participants, their data, or biological material

Policy information about studies with human participants or human data. See also policy information about sex, gender (identity/presentation), and sexual orientation and race, ethnicity and racism.

Reporting on sex and gender	*Use the terms sex (biological attribute) and gender (shaped by social and cultural circumstances) carefully in order to avoid confusing both terms. Indicate if findings apply to only one sex or gender; describe whether sex and gender were considered in study design; whether sex and/or gender was determined based on self-reporting or assigned and methods used. Provide in the source data disaggregated sex and gender data, where this information has been collected, and if consent has been obtained for sharing of individual-level data; provide overall numbers in this Reporting Summary. Please state if this information has not been collected. Report sex- and gender-based analyses where performed, justify reasons for lack of sex- and gender-based analysis.*
Reporting on race, ethnicity, or other socially relevant groupings	*Please specify the socially constructed or socially relevant categorization variable(s) used in your manuscript and explain why they were used. Please note that such variables should not be used as proxies for other socially constructed/relevant variables (for example, race or ethnicity should not be used as a proxy for socioeconomic status). Provide clear definitions of the relevant terms used, how they were provided (by the participants/respondents, the researchers, or third parties), and the method(s) used to classify people into the different categories (e.g. self-report, census or administrative data, social media data, etc.) Please provide details about how you controlled for confounding variables in your analyses.*
Population characteristics	*Describe the covariate-relevant population characteristics of the human research participants (e.g. age, genotypic information, past and current diagnosis and treatment categories). If you filled out the behavioural & social sciences study design questions and have nothing to add here, write "See above."*
Recruitment	*Describe how participants were recruited. Outline any potential self-selection bias or other biases that may be present and how these are likely to impact results.*
Ethics oversight	*Identify the organization(s) that approved the study protocol.*

Note that full information on the approval of the study protocol must also be provided in the manuscript.

Field-specific reporting

Please select the one below that is the best fit for your research. If you are not sure, read the appropriate sections before making your selection.

[x] Life sciences [ ] Behavioural & social sciences [ ] Ecological, evolutionary & environmental sciences

For a reference copy of the document with all sections, see nature.com/documents/nr-reporting-summary-flat.pdf

Life sciences study design

All studies must disclose on these points even when the disclosure is negative.

Sample size	For RNA analysis, three independent biological samples were analyzed, and each sample was collected from 12 plants. For bacterial pathogen growth assessment, three leaves on each plant were inoculated and a total of 12 plants were used for each genotype/treatment. Eight to twenty leaves per genotype/treatment were collected to examine the growth of the pathogen. A leaf disc was collected from each leaf and used as an independent biological sample.
Data exclusions	No data were excluded from the analyses.
Replication	Pathogen growth experiments were repeated at least three times with similar results. NAD(P) and eNAD(P) measurement experiments were repeated two to three times with similar results. qPCR experiments were repeated twice with similar results.
Randomization	For RNA sample collection, one leaf from each of the 12 plants was collected, cut into small pieces, and mixed, then 100 mg leaf tissues were taken from the mixture for RNA extraction. For pathogen growth assessment, eight to twenty leaves were randomly taken from a total of 36 leaves. For eNAD(P) measurement, 12 leaves from 12 plants were collected for apoplastic washing fluid collection. Total NAD(P) were measured using 100 mg leaf tissues from eight leaves.

Blinding	Blinding was not possible for the described experiments, as the investigator had to carefully label each genotype/treatment to ensure that samples were not messed up.

Reporting for specific materials, systems and methods

We require information from authors about some types of materials, experimental systems and methods used in many studies. Here, indicate whether each material, system or method listed is relevant to your study. If you are not sure if a list item applies to your research, read the appropriate section before selecting a response.

Materials & experimental systems

n/a	Involved in the study	
☒	☐	Antibodies
☒	☐	Eukaryotic cell lines
☒	☐	Palaeontology and archaeology
☒	☐	Animals and other organisms
☒	☐	Clinical data
☒	☐	Dual use research of concern
☐	☒	Plants

Methods

n/a	Involved in the study	
☒	☐	ChIP-seq
☒	☐	Flow cytometry
☒	☐	MRI-based neuroimaging

Dual use research of concern

Policy information about dual use research of concern

Hazards

Could the accidental, deliberate or reckless misuse of agents or technologies generated in the work, or the application of information presented in the manuscript, pose a threat to:

No	Yes	
☒	☐	Public health
☒	☐	National security
☒	☐	Crops and/or livestock
☒	☐	Ecosystems
☒	☐	Any other significant area

Experiments of concern

Does the work involve any of these experiments of concern:

No	Yes	
☒	☐	Demonstrate how to render a vaccine ineffective
☒	☐	Confer resistance to therapeutically useful antibiotics or antiviral agents
☒	☐	Enhance the virulence of a pathogen or render a nonpathogen virulent
☒	☐	Increase transmissibility of a pathogen
☒	☐	Alter the host range of a pathogen
☒	☐	Enable evasion of diagnostic/detection modalities
☒	☐	Enable the weaponization of a biological agent or toxin
☒	☐	Any other potentially harmful combination of experiments and agents

Plants

Seed stocks	The following seeds were purchased from Arabidopsis Biological Resource Center: fin4-3 (SAIL_1145_B10), fmo1 (SALK_026163), and lecrk-VI.2-2 (SAIL_1146_B02). The rbohD and rbohF seeds were obtained from Dr. Jeffery Dangl (University of North Carolina at Chapel Hill), the bak1-5 bkk1 seeds were provided by Dr. Libo Shan (Texas A&M University), and the 35S:CD38 transgenic lines were previously made in the lab.
Novel plant genotypes	The 35S:FIN4/fin4-3, Dex:FIN4/fin4-3, and Dex:LecRK-VI.2/lecrk-VI.2 transgenic lines were made in this study through Agrobacterium-mediated transformation. Two independent transgenic lines were analyzed and T4 generation seeds were used for the experiments.
Authentication	The fin4-3 mutant was confirmed by reduced NAD(P) levels and was complemented with a 35S:FIN4 transgene. The fmo1, rbohD, and rbohF mutants were confirmed by PCR and their specific phenotypes (fmo1 has no SAR and rbohD and rbohF accumulate reduced ROS upon pathogen infection). The inducibility of the Dex:FIN4/fin4-3 and Dex:LecRK-VI.2/lecrk-VI.2 were tested by both GUS staining and qPCR.
