# Supplementary material for: N-hydroxypipecolic acid triggers systemic acquired resistance through extracellular NAD(P)
Source: Nat Commun. 2023 Oct 27;14:6848. doi: 10.1038/s41467-023-42629-0 (PMC10611778; doi:10.1038/s41467-023-42629-0)
Supplement: Supplementary file 4 — Description of Additional Supplementary Files [file 41467_2023_42629_MOESM4_ESM.pdf]

Description of Additional Supplementary Files

Supplementary Data 1: Differentially Expressed Genes (DEGs) in the Systemic Leaves of Wild-Type (WT) and *fin4-3* Plants at 48 hr after SAR Induction

Supplementary Data 2: SAR-Related DEGs in the Systemic Leaves of Wild-Type (WT) and *fin4-3* Plants at 48 hr after SAR Induction
